# Supplementary material for: Emergence of cooperative bistability and robustness of gene regulatory networks
Source: PLoS Comput Biol. 2020 Jun 29;16(6):e1007969. doi: 10.1371/journal.pcbi.1007969 (PMC7351242; doi:10.1371/journal.pcbi.1007969)
Supplement: S1 Fig — All the GRNs which act as toggle switches and have no lethal edge in the fittest ensemble are shown. Blue lines indicate activation, and red lines indicate repression. The interaction matrices for these GRNs are available at Zenodo (DOI: 10.5281/zenodo.3716026). (PDF) [file pcbi.1007969.s001.pdf]

S1 Fig. All GRNs classified as "toggle switch without lethal edge". All the GRNs which act as toggle switches and have no lethal edge in the fittest ensemble are shown. Blue lines indicate activation, and red lines indicate repression. The interaction matrices for these GRNs are available at Zenodo (DOI: 10.5281/zenodo.3716026).

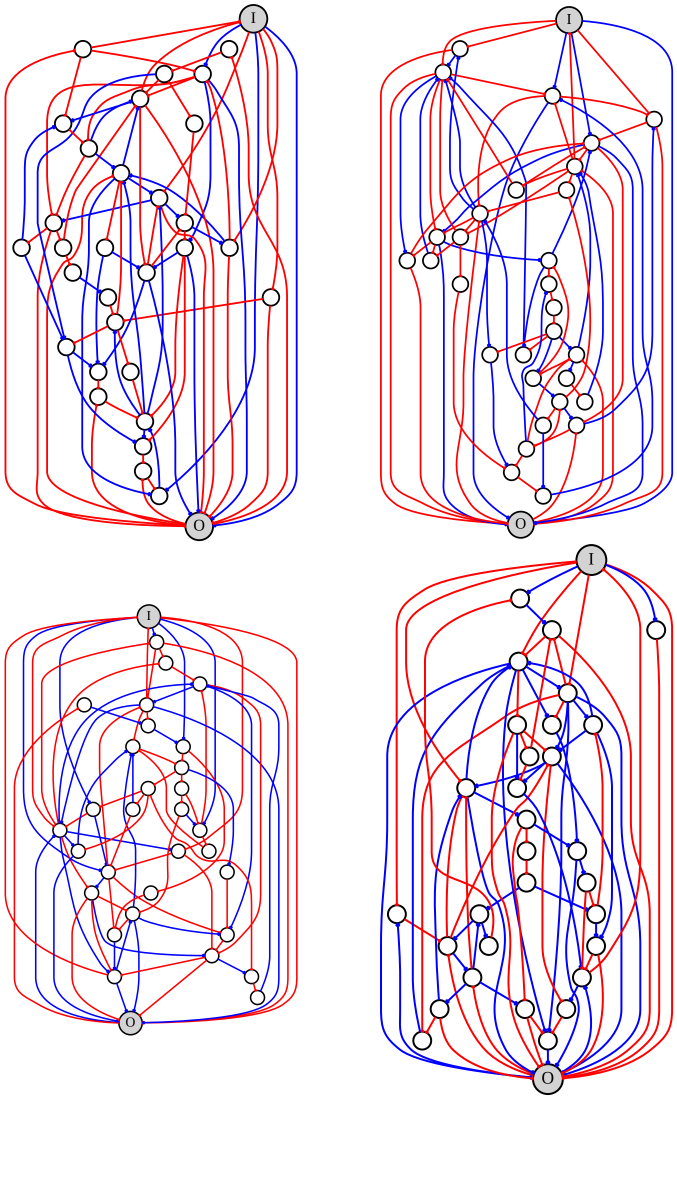

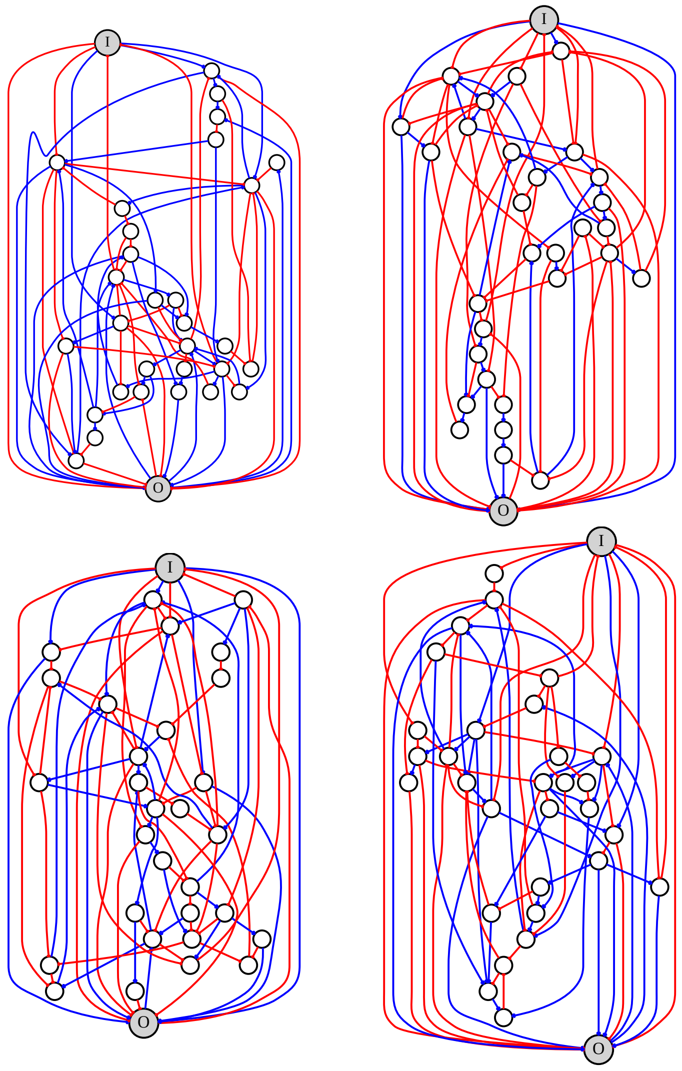
I
O

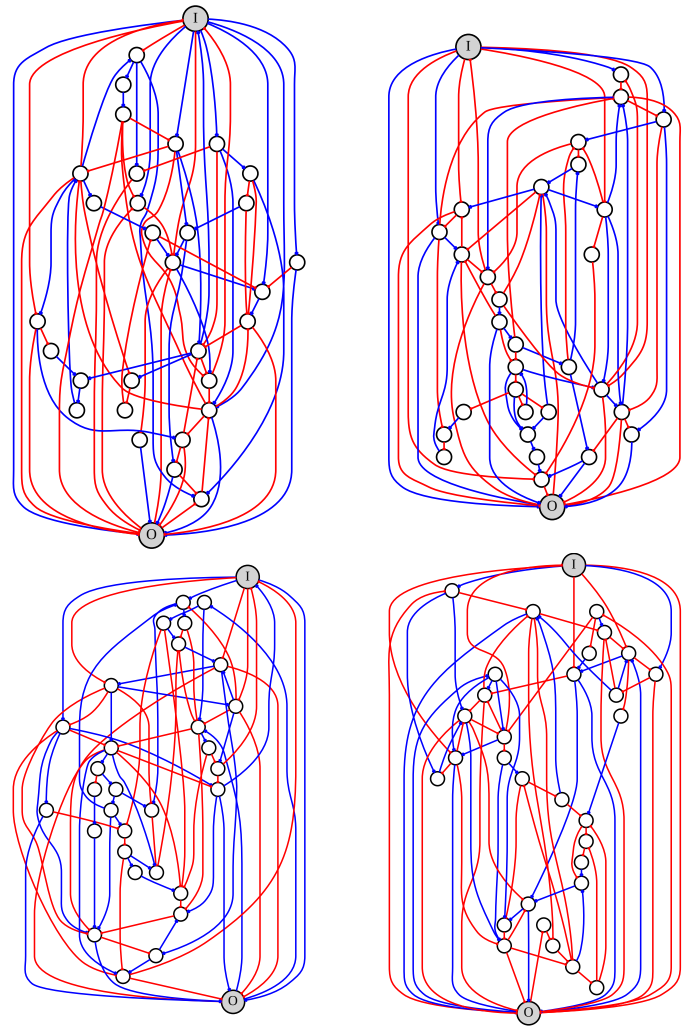
I
O
I
O
I
O
I
O

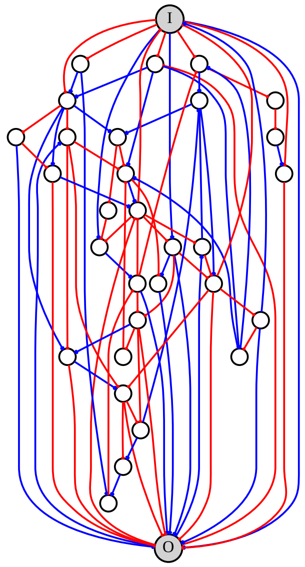
I
O
