# Supplementary material for: Emergence of cooperative bistability and robustness of gene regulatory networks
Source: PLoS Comput Biol. 2020 Jun 29;16(6):e1007969. doi: 10.1371/journal.pcbi.1007969 (PMC7351242; doi:10.1371/journal.pcbi.1007969)
Supplement: Attachment — Submitted filename: Response_to_Reviewers.pdf [file pcbi.1007969.s002.pdf]

Reply to editor

Thank you very much for carefully reading the manuscript. We considered the reviewers' comments fully and revised the manuscript accordingly. Please read the reply to the reviewers. We hope the revision was appropriately made and satisfy the reviewers' requirement.

Macoto Kikuchi

Reply to reviewer 1

In this manuscript, the authors apply MC sampling to GRN fitness landscape. The model structure, its rationale, the methodology to sample models, and the results, are all well described. Some interesting types of behaviours of GRNs are displayed, including the toggle-switch-response, and noise induced response. This type of work helps improve our understanding and is an important complementary type of work compared with simulations of specific models for GRNs. My comments are only minor:

Thank you for carefully reading the manuscript and valuable comments. We revised the manuscript according to your suggestions. Here we reply to each of your comments.

1) Code used for the sampling and model simulations seems not to be available. It would be useful to do so.

We published codes used for sampling and analysis at Zenodo as DOI: 10.5281/zenodo.3716026. All the data used in the study are also made available there. We added a "Source codes and data availability" subsection after the "Method" subsection as follows:

> The source codes and the obtained data sets are available at Zenodo (DOI: 10.5281/zenodo.3716026).

2) Similarly for specific examples of GRNs with e.g. high fitness.

We provided structures of all GRNs classified as "toggle switch without a lethal edge" (TSwoLE) in the supplemental material "S1 Figure". We also made structural data of these GRNs available at Zenodo stated above.

3) It would useful if a bit more specific connections could be made to experimental evidence for some of the properties that are discussed. I understand results with these simple networks cannot be directly tested but for some of the suggestions that can be based on this work, data might be available in other labs/literature.
4) Related to this, it should also be possible to make an explicit comparison between the networks studied here, and available known GRNs from various organisms. Can anything be said about how similar/dissimilar the most fit networks in this study, and GRNs from various organisms, are?
5) Similarly, a bit more explicit comparison with literature would be warranted. Other studies where fitness landscapes of GRN are sampled or somehow analyzed (without applying evolutionary approaches) are mostly not used.
6) Is it possible to further analyze the selected GRNS (e.g. the most fit ones) to further get insight into properties of such 'best networks'? E.g. does the presence/absence of certain network sub-motifs correlate with fitness or with other properties of interest?

Although it is not straightforward to compare our results with those of experimental studies and other numerical studies, we added some qualitative comparisons.

1. Almost all GRNs in the fittest ensemble exhibit bistability. In the revised manuscript, we classified them into three: toggle switch, one-way switch, the unswitchable. GRNs of the former two classes are observed in biological GRNs. We cited the phage λ and the Xenopus egg as examples of toggle switch GRNs and cited the MAPK cascade working in the maturation of the Xenopus oocyte as an example of the one-way switch. While in system biology a small number of components have been taken into account for explaining their function, our results indicate a possibility that more genes participate in realizing bistability.

   We added the following paragraph to the "Emergence of bistable responses" subsection in the "Results" section:

   > The bistable GRNs can be classified into three classes according to the range where the bistability occurs. The first is the toggle switch. For the GRNs belonging to this class, the GRNs are monostable both at $I = 0$ and 1 and the bistable range lies between them. The second is the one-way switch [44]. In this class, the bistable range includes either $I = 0$ or 1. In the third class, both $I = 0$ and 1 are included in the bistable range. Since GRNs belonging to this last class do not work as a switch, we call this class as "unswitchable". We found that among 4524 bistable GRNs, the ratio of the toggle switch, the one-way switch, and the unswitchable were 27.8%, 42.5%, and 29.7%, respectively. The GRNs belonging to the one-way switch and the unswitchable were expected not to follow the abrupt change of the input, at least in one direction. By running the dynamics setting I as 0 for the first 1000 steps, then 1 for the next 1000 steps, and 0 again for the last 1000 steps, we confirmed that the toggle switch GRNs followed the input change, while other GRNs were unable to follow the input change properly due to them being trapped by the wrong fixed point.

We also added the following paragraph to the "Discussion" section:

> There are many examples of toggle switches and one-way switches. One of the well-known toggle switches is found in the lysogenic-lytic transition of the phage λ [25,26,65,72]. The cdk1 activation system of the Xenopus egg is another example, which behaves as a reversible bistable switch having a clear hysteresis in response to cyclin B1 concentration [53,56,68]. One-way switches are utilized in GRNs related to cell-fate decisions. A widely-analyzed example is the maturation of the Xenopus oocyte, which is regulated by the bistability of the MAPK cascade [48]. However, a relatively small number of components were considered for discussing the mechanism of bistability in most cases. In contrast, the bistable GRNs we found in this study consisted of a large number of genes. The present result indicates a possibility that more genes than considered participate to realize bistability in biological systems.

2. Following your suggestion, we conducted the motif analysis for GRNs in the fittest ensemble. There, we restricted ourselves to analyze triangular motifs consisted of three nodes and three edges. As a result, we found that the coherent feedforward loops (+FFL) are the most abundant, and the positive feedback loops (+FBL) are the next abundant. While the abundance of +FFL is consistent with the observations for *E. Coli* and yeast. the incoherent feedforward loop, which is also abundant in biological GRNs, were scarce in our model.
   +FBL is known to be important in multistable GRNs. In particular, the combination of double-negative FBL and self-activation is observed in, for example, the phage λ or GATA1-PU.1 system. Burda *et al.*, who conducted a random sampling of GRNs without using evolutionary simulations, found that this combination is ubiquitous in multistable GRNs. Although this structure is prohibited in our model, the +FBL abundance is consistent with these studies and we consider that equivalent structures were formed using +FBLs in our model.
   We added the "Motif analysis" subsection to the "Result" section as follows:

   > Network motifs are defined as "patterns of interconnections that recur in many different parts of a network at frequencies much higher than those found in randomized networks" [27,45]. We investigated the distributions of motifs consisting of three nodes and three edges. Since self-regulation and the mutual regulation are prohibited in our model, three nodes and three edges form a triangular loop. Such loops are classified into two classes: the feedback loops (FBL) and the feedforward loops (FFL). FBL is classified further into the positive FBL (+FBL) and the negative FBL (-FBL). The former includes an even number of repressions and the latter includes an odd number of repressions. Similarly, FFL is classified further into the coherent FFL (+FFL), which includes an even number of repressions, and the incoherent FFL (-FFL), which includes an odd number of repressions.

For the GRNs in the fittest ensemble for $N = 32$ and $C = 5$, we counted the number of these loops. Fig.9a shows the number distributions of the four types of loops. As a reference, we also show the results for $f \in [0, 0.01]$ (Fig.9b), which can be regarded as the random ensemble of GRNs. For the random ensemble, the distributions of +FBL and -FBL agreed with each other as expected and those for +FFL and -FFL agreed with each other as well. The fittest ensemble exhibited a different tendency. The loop that was observed most frequently was +FFL, and the next was +FBL. These two types of loops were significantly abundant compared to the random ensemble and thus were considered to be motifs. In contrast, -FBL and -FFL were scarce compared to the random ensemble.

We also added the following paragraph on motif analysis to the "Discussion" section, where we mentioned the numerical work by Burda *et al.* in addition to experimental works.

We analyzed the network motifs consisting of three edges for GRNs in the fittest ensemble. We found that the motif with the largest abundance was the coherent feedforward loop (+FFL) and the next was the positive feedback loop (+FBL). In real GRNs, FFLs are the most frequently observed motifs [27]. Shen-Orr *et al.* reported that +FFL is a characteristic motif in the GRN of *E. Coli* [45]. Later studies revealed that the incoherent feedforward loop (-FFL) is the next abundant motif [74]. Similar observations were also obtained for yeast [27,75]. The result of the present study agrees with these observations in part because +FFL was the most abundant. In contrast, -FFL was rather scarce in our model. +FFL is known to generate a sign-sensitive delay [27]. However, since we considered only the steady-state response in the definition of fitness, such delay may not be important. To explain +FFL abundance, we assumed that it is utilized for creating a large response from a "sloppy" response function [11].

The second abundant motif, +FBL, generates bistability when combined with ultrasensitive responses [42,48,51,57,69,70,73,76]. Typical structures are the double-negative FBL and the double-positive FBL. Burda *et al.* investigated the motifs of functional GRNs by randomly generating GRNs using the Markov chain Monte Carlo method [22]. They found that a combination of double-negative FBL and self-activation is ubiquitous in GRNs exhibiting multistability. Such a structure is found in phage λ [25,26] and is utilized for realizing bistability. This structure works as a bistable switch rather robustly, even when the kinetic parameters are randomly changed [77]. It is also known to have a potential for exhibiting tristability, observed, for example, in the GATA1–PU.1 system [78,79]. In contrast, since mutual regulation and self-regulation are prohibited in our model, such a structure is not possible. However, the +FBL abundance suggests that +FBLs are needed to exhibit bistability. We consider that structures playing equivalent roles to the double-positive FBL are formed by several combinations of +FBLs. Conversely, since some GRNs in the fittest ensemble lack -FFL and/or -FBL, they are not mandatory for bistability. Our result that particular motifs overexpress as the fitness becomes high is consistent with the finding by Burda *et al.*, wherein a motif appropriate

for function emerges automatically. For understanding the roles of motifs, we require a more detailed analysis based on a larger number of samples.

3. On the mutational robustness, we noticed a rewiring experiment by Isaran *et al.*. To compare with their experiment, we conducted a single-edge addition experiment comprehensively to GRNs belonging to "toggle switch without a lethal edge. We found that these GRNs were robust for most of the single-edge addition. The result is consistent with the rewiring experiment.
   We added the following paragraph to the "Robustness against mutation" subsection in the "Result" section:

   > Among these 17 GRNs, 13 were toggle switches and the rest four were one-way switch. This ratio of the toggle switches is significantly higher than that for all the GRNs in the fittest ensemble. We show the network structures of all 13 such GRNs in S1 Figure. We call them "toggle switches without a lethal edge" (TSwoLE). For TSwoLE, we studied the effects of another type of mutation, namely, the addition of a single edge. We tried all the possible single-edge additions for each GRN. We found that all the GRNs kept high fitness for more than 95% of possible single-edge additions. In contrast, this ratio differs largely from network to network for other GRNs belonging to the fittest ensemble. Thus, TSwoLE are particularly robust to mutation.

   And we added the following paragraph to the "Discussion" section:

   > Isaran *et al.* reported a rewiring experiment for GRN of *E. Coli*, namely, they added new regulatory interactions to GRNs [83]. They reported that the cells were robust for most cases of rewiring. To compare with this experiment, we made comprehensive single-edge addition to the obtained GRNs classified as the "toggle switches without a lethal edge" (TSwoLE) and confirmed that they are robust for most of such mutations. That is, these GRNs are robust both for single-edge deletion and single-edge addition. This result is consistent with the rewiring experiment. Although such GRNs are a minority among the fittest ensemble, they are not so scarce. Robustness for edge addition is advantageous in acquiring new functions and is considered as a possible source of evolvability.

4. Also for the mutational robustness, we added a discussion on the results of Ciliberti *et al.* using a random sampling method. Although comparison with our results is not straightforward, their observation that the functional GRNs form a large cluster in the neutral space seems not inconsistent with our result that GRNs in the fittest ensemble are robust for single-edge deletion.
   We added the following paragraph to the "Discussion" section.

   > Ciliberti *et al.* conducted a numerical experiment in which GRNs were sampled randomly [19]. They found that the functional GRNs formed a large cluster in the neutral space. Although

exploring the structure of the neutral space from our result is not straightforward, the fittest ensemble is close in concept to the neutral space, and the fact that many edges are neutral for deletion indicates that the robust GRNs are not isolated in the fittest ensemble.

5. We found we had missed papers by Burda *et al.* and Zagorski *et al.* both of which deeply related to our work from the viewpoint of methodology. We add the following sentences to the "Introduction" section:

   In studies by Refs.[22,23], the Markov Chain Monte Carlo method (Metropolis method) was used to sample functional GRNs randomly, for which the network motifs were analyzed. This method is suitable for efficient sampling of functional GRNs.

7) page 7 mentions "An inverse- λ distribution". It is not clear to me what this means.

We meant that as fitness increases a minor peak with smaller f' started to appear and its position went lower with the fitness. The expression "inverse-λ distribution" described the shape of the light blue region enclosed by a scarlet line seen in the following figure:

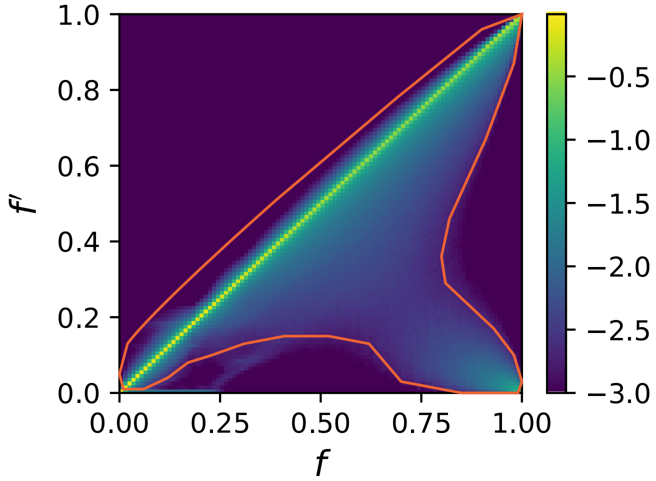

We altered the description as follows:

Most of f's did not differ largely from the original fs. Thus, the fitness did not change much after the single-edge deletion. However, the edges for which the fitness dropped significantly when deleted started to appear near $f \simeq 0.6$. As f increased, the low peak corresponding to such edges (seen as white area) moved to $f' \simeq 0$ and the edges with intermediate f' decreased.

Reply to reviewer 2

In the present manuscript, Nagata and Kikuchi explored the fitness landscape of gene regulatory networks (GRNs) and analyzed the emergence of robustness. They considered a toy model of GRNs with one input gene and one output gene, and utilized the multicanonical Monte Carlo method to classify the GRNs per their fitness. They showed that GRNs can acquire bistability to distinguish between the two different input states ' ON ' and ' OFF ' as the fitness increases. As the bistable GRNs are robust again noise, the robustness of GRNs can be regarded as a byproduct of high fitness. Overall, the study has been well designed and performed.

Regarding the robustness of GRNs, there is a hypothesis that the dynamics behaviors of GRNs are mainly determined by the topologies of the GRNs. The following relevant papers should be appropriately cited and discussed.

Cell. 2009 Aug 21;138(4):760-73. doi: 10.1016/j.cell.2009.06.013.

PLoS Comput Biol. 2017 Mar 31;13(3):e1005456. doi: 10.1371/journal.pcbi.1005456

BMC Syst Biol. 2018 Jun 19;12(1):74. doi: 10.1186/s12918-018-0594-6.

Regarding the bistability or multi-stability of GRNs, the authors may want to discuss more biological examples, such as the genetic toggle switch GATA-1/PU.1 governing lineage differentiation. The following relevant studies should be appropriately cited and discussed.

Dev Biol. 2007 May 15;305(2):695-713.

Phys Biol. 2017 May 23;14(3):035007. doi: 10.1088/1478-3975/aa6f90.

Thank you for carefully reading the manuscript and valuable comments. According to your suggestion, we read all the suggested papers, and determined that the followng three are relevant to our work:

- PLoS Comput Biol. 2017 Mar 31;13(3):e1005456. doi: 10.1371/journal.pcbi.1005456
- Dev Biol. 2007 May 15;305(2):695-713.
- Phys Biol. 2017 May 23;14(3):035007. doi: 10.1088/1478-3975/aa6f90.

We added the following sentences to the "Discussion" section.

> This structure works as a bistable switch rather robustly, even when the kinetic parameters are randomly changed [77]. It is also known to have a potential for exhibiting tristability, observed, for example, in the GATA1–PU.1 system [78,79].

Reply to revewer 3

This interesting manuscript presents a prototypical model of a gene regulatory network that responds to a signal. A node of this network is designated as output and the difference between the expression of this node in the two extreme values of the signal (0 and 1) is defined as the network' s fitness. The manuscript shows that the overwhelming majority of the networks in the highest fitness category are bistable (they have two fixed points for certain values of the signal). Moreover, these high-fitness networks are robust to noise, and even exhibit noise-induced response, i.e. the presence of noise in the perceived value of the signal or of internal nodes helped the system respond to a 0-1-0 pattern in the signal. These networks also exhibit significant resilience to mutations in edges or nodes. The manuscript suggests that robustness against noise or mutation is a byproduct of high fitness, and may be readily acquired through evolution.
I think these suggestions are intriguing and the manuscript will be a valuable contribution to the literature if a number of unclear points are addressed.

Thank you for carefully reading the manuscript and valuable comments. We revised the manuscript according to your suggestions. Here we reply to each of your comments.

1. The introduction starts with presenting "function" as a foundational concept and characteristic of living systems. It is at this point that the embodiment of function assumed in this work needs to be clearly presented and justified. There also should be some (later) discussion on the dependence of the conclusions on the assumed embodiment of function. For example, the signal is assumed to be continuous and in the range [0,1], yet the assumed function depends on the difference in response to the two extreme values. The bistable, high fitness networks have low sensitivity to smaller changes in the signal. Is that acceptable biologically?

We agree that the explanation was confusing. The function we required for the GRNs was to distinguish two different inputs, ”on” and ”off”, sharply and thus the inputs are assumed to be two-valued. However, to investigate the dynamical properties of obtained GRNs, we tried to vary input value continuously and confirmed the bistability.

To state the function more clearly, we added the following sentence to the definition of the function in the ”Model” subsection.

> This is the functionality assumed for GRNs in this study.

We also added the following sentences to the beginning of the ”Emergence of cooperative bistability” subsection in the ”Results” section to make it clear that we tried intermediate input values to investigate the dynamical properties of GRN apart from the originally required function:

> Although the function we required for GRNs was to discriminate $I = 0$ and 1 sharply, to investigate

the dynamical properties of highly-fit GRNs, we studied how the steady-state response changed at intermediate values of input.

As you pointed out, bistable GRNs work as a low-pass filter and are insensitive to a small change of input. This is the origin of robustness for the noise. Such bistable GRNs have been found in many biological GRNs and the bistability is considered to be effective for stable gene expressions. The bistable GRNs we obtained are classified into three classes: toggle switch, one-way switch and the unswitchable. Among them, the toggle switch GRNs are found in, for example, the phage λ and the Xenopus egg. The one-way switch GRNs are utilized for cell-fate decisions; a representative example is the MAPK cascade in the Xenopus oocyte maturation. Thus, some of the bistable GRNs are considered to be biologically relevant. In contrast, the unswitchable may not have a biological meaning.

To clarify this, we added the following paragraph to the Emergence of cooperative bistability" subsection in the "Results" section:

> The bistable GRNs can be classified into three classes according to the range where the bistability occurs. The first is the toggle switch. For the GRNs belonging to this class, the GRNs are monostable both at $I = 0$ and 1 and the bistable range lies between them. The second is the one-way switch [44]. In this class, the bistable range includes either $I = 0$ or 1. In the third class, both $I = 0$ and 1 are included in the bistable range. Since GRNs belonging to this last class do not work as a switch, we call this class as "unswitchable". We found that among 4524 bistable GRNs, the ratio of the toggle switch, the one-way switch, and the unswitchable were 27.8%, 42.5%, and 29.7%, respectively. The GRNs belonging to the one-way switch and the unswitchable were expected not to follow the abrupt change of the input, at least in one direction. By running the dynamics setting I as 0 for the first 1000 steps, then 1 for the next 1000 steps, and 0 again for the last 1000 steps, we confirmed that the toggle switch GRNs followed the input change, while other GRNs were unable to follow the input change properly due to them being trapped by the wrong fixed point.

We also added the following paragraph mentioning biological examples to the "Discussion" section:

> There are many examples of toggle switches and one-way switches. One of the well-known toggle switches is found in the lysogenic-lytic transition of the phage λ [25,26,65,72]. The cdk1 activation system of the Xenopus egg is another example, which behaves as a reversible bistable switch having a clear hysteresis in response to cyclin B1 concentration [53,56,68]. One-way switches are utilized in GRNs related to cell-fate decisions. A widely-analyzed example is the maturation of the Xenopus oocyte, which is regulated by the bistability of the MAPK cascade [48].

2. The abstract refers to “switching the fixed points” and emergence of new phenotypes. Even after reading the manuscript twice this is not clear to this reviewer. Probably what is needed here is an intuitive way to refer to bistability.

When fitness was low, GRNs were monostable in the whole range of the input, which means that

GRNs have a single fixed point, which continuously changes with the value of input. In contrast, since GRNs with high fitness were bistable in an intermediate range of input between ”on” and ”off”, it is suggested that such GRNs have two fixed points corresponding to two distinct inputs. Although we called the emergence of such bistability as a ”new phenotype”, we agree that this expression was confusing. Accordingly, we altered the sentence pointed out as follows:

> (1)Highly-fit GRNs exhibited bistability for intermediate input between ”on” and ”off”. This means that such GRNs responded to two input states by using different fixed points of dynamics. This bistability emerges necessarily as fitness increases.

> 3. Two criteria in network construction seem to be partially contradictory. First (line 79) paths must exist from all the nodes to the output node. Second (line 126), the node that exhibited the largest sensitivity except the input node was selected as the output node. What was done if the first criterion was not satisfied for node that exhibited the largest sensitivity?

We added the following sentence to the ”Model” subsection to clarify that when the node of the largest sensitivity did not match the criterion, such GRN was discarded.

> If the node of the largest sensitivity lacked paths from one or more other genes, such GRN was not used.

> 4. The decision to prohibit self-regulation and mutual regulation of two genes should be motivated a bit more than “for the sake of simplicity” and “do not cause any problem”. Perhaps the reason was a wish to avoid known/expected sources of bistability so that the results uncover less known or unexpected sources.

A combination of mutual repression and self-activation is known to give rise to bistability. In our model, we prohibited such combination to be formed. To clarify this, we added the following sentences to the ”Model” subsection:

> We prohibited both the mutual regulation of two genes and the self-regulating loop. They are frequently observed in real GRNs and in particular, the combination of mutual repression and self-activation is known to give rise to bistability. Thus, this restriction means that we excluded a representative structure of bistable GRNs.

We also conducted preliminary computations for the case that these two types of regulations were permitted. We found that the results were qualitatively unchanged. The following figures show the results corresponding to Fig.2 and Fig.4b for $N = 32$ and $C = 6$.

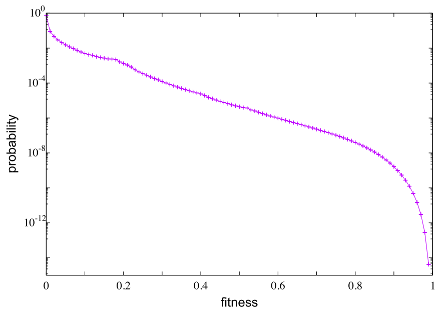

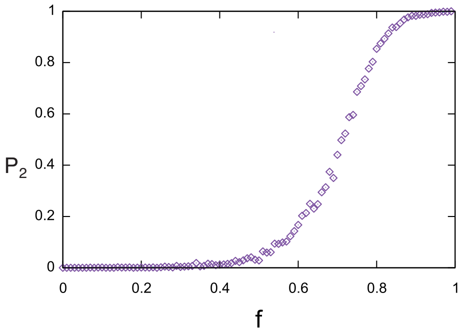

Although we did not show these figures in the revised manuscript, we added the following sentence to the "Model" subsection:

> We conducted preliminary computations for the model that these two types of regulations are permitted and confirmed that the results were qualitatively unchanged.

5. The explanation for choosing a tanh function instead of a Hill function (in line 104) is not sufficient. Perhaps the reason has to do with having to choose a Hill coefficient value in the latter case? What would change if a Hill function with relatively large Hill coefficient were used?

The response function we used is equivalent to a widely used sigmoidal function

$$R(x) = \frac{1}{1 + e^{-a(x-b)}}$$

with a particular choice of parameter: $a = 1$ and $b = 0$. This function also resembles to the response function proposed by Mjolsness inspired by Hill function; A "shifted" Hill function

$$R'(x) = \frac{1}{1 + \left(\frac{x+a}{k}\right)^n}$$

is close to our response function, as shown in the following figure, where the best-fit parameters are chosen:

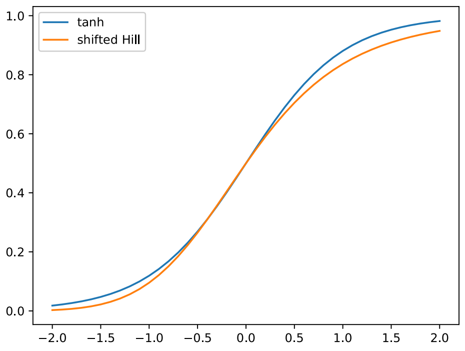

We applied this $R'(x)$ to GRNs in the fittest ensemble and found that 97% of GRNs kept high fitness. Thus, we consider that the results do not depend largely on the details of the response function.

We also conducted preliminary computations using steeper response functions, which correspond to one with larger Hill coefficient. Although the detail of the fitness landscape changed, we found that the results were qualitatively unchanged. The following figure shows the fitness landscape for $R(x) = (\tanh 2x + 1)/2$.

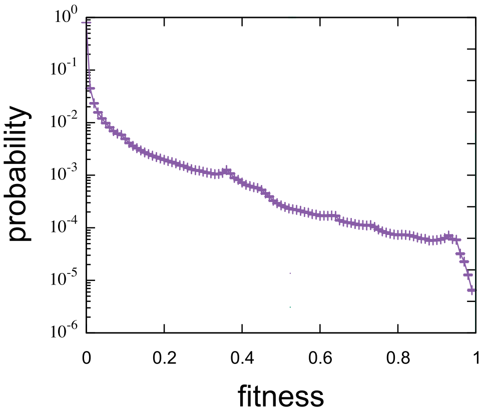

In the revised manuscript, we altered the response function to

$$R(x) = \frac{1}{1 + e^{-a(x-b)}}$$

and changed the description as follows:

> The discontinuous sign function is sometimes used for the response function $R(x)$ with x being assumed to take ± 1 [4,5,20]. But since we want to define a fitness function having a continuous value, we used the above sigmoidal function, which reflects the fact that the responses in living systems are imprecise and stochastic (or "sloppy" according to Ref.11).
>
> This type of response function was proposed in Ref.4 and was frequently used [6,10,11,28,29]. The parameter a determines the steepness of the sigmoidal function and b gives the threshold. In this paper, we fixed the parameters to $a = 1$ and $b = 0$. In this case, $R(x)$ is equivalent to $(\tanh x + 1)/2$ [8]. We also conducted preliminary computations for a steeper sigmoidal function and found that the results are qualitatively similar. The function is also close to the one proposed by Mjorsness inspired by the Hill function [24]. Each node exhibits the spontaneous expression $x_i = 0.5$ even when no input is provided. We also performed preliminary computation for steeper function such as $(\tanh 2x + 1)/2$, which corresponds to a response function with a larger Hill coefficient, and confirmed that results were qualitatively unchanged.

6. The sentence in lines 110- 113 seems important but it is not clear. Why do the levels of expression decay from one gene to the next? What exactly do you mean by feed-forward type regulations? Maybe you mean a feed-forward loop, which is the meeting of shorter and longer paths in the same target node?

Yes, we meant the feed-forward loops. We expected feed-forward loops played an important role in transferring input information to output. Following a suggestion by another reviewer, we conducted the motif analysis and found that feed-forward loops were indeed abundant. So we deleted the mention on the feed-forward loop from the "Model" subsection and added the "Motif analysis" subsection to the end of the "Results" section. We also added the following discussion on feed-forward loops to the "Discussion" section.

We analyzed the network motifs consisting of three edges for GRNs in the fittest ensemble. We found that the motif with the largest abundance was the coherent feedforward loop (+FFL) and the next was the positive feedback loop (+FBL). In real GRNs, FFLs are the most frequently observed motifs [27]. Shen-Orr *et al.* reported that +FFL is a characteristic motif in the GRN of *E. Coli* [45]. Later studies revealed that the incoherent feedforward loop (-FFL) is the next abundant motif [74]. Similar observations were also obtained for yeast [27,75]. The result of the present study agrees with these observations in part because +FFL was the most abundant. In contrast, -FFL was rather scarce in our model. +FFL is known to generate a sign-sensitive delay [27]. However, since we considered only the steady-state response in the definition of fitness, such delay may not be important. To explain +FFL abundance, we assumed that it is utilized for creating a large response from a "sloppy" response function [11].

7. In lines 236-238, what is the intuitive meaning of the case that the region of bistability includes I=0, I=1, or both? Which of the three cases is referred to as one-way switch?

If the range of bistability does not include both I=0 and 1, the GRN is classified as the toggle switch. If the range of bistability includes one of either I=0 or 1, the GRN is classified as the one-way switch. Adding to them, we introduced a new category "the unswitchable" in which the range of bistability includes both I=0 and 1. To explain more clearly, we extended the description on the classification of the bistable GRNs as the paragraph quoted in the reply to your question 1.

More minor points:

1. Please check the description that introduces gene regulatory networks to avoid the misinterpretation that genes interact with each other or regulate each other. It needs to be said clearly at least once that gene products, chiefly proteins, interact with each other. For example, transcription factor proteins regulate the transcription of genes into mRNA. Response to the environment that ultimately leads to gene regulation also involves protein-protein interactions, miRNA- RNA interactions and reactions. The framework can plausibly be used in all of these cases so there is no need for a restrictive "gene" language.

We added the following description of the mechanism of gene regulation in the "Introduction" section:

GRNs are complex networks of genes that mutually regulate their expression levels to adapt the cell state to environmental conditions. The regulation mechanism of GRNs is as follows: A protein called a transcription factor (TF) is expressed from one gene. This TF acts either as the activator or the repressor of other genes. The activator binds to an upstream region of the promoter of the target gene and promotes its expression by recruiting the RNA polymerase, which produces mRNA. A protein is synthesized at a ribosome according to the mRNA sequence. A gene is sometimes expressed without the activator owing to spontaneous binding of the RNA polymerase. In contrast, the repressor binds to the promoter of the target gene to block RNA polymerase. As

a result, the expression of the target gene is prevented. The produced protein then acts as a TF to yet other genes, and thus the genes regulate each other.

2. In line 43, "fitness is taken as a parameter instead of the genotype". One would rarely consider genotype a parameter. Maybe you meant "variable", not "parameter", for both cases?

As you pointed out, the word "parameter" was not appropriate. We meant "variable". We changed the sentence as follows

> We explored a different type of a fitness landscape, in which the fitness was considered as an independent variable.

3. In line 57, "model we dealt with" would be better as "model we constructed"

We changed the sentence according to your advice.

4. In line 63, "to the direction" should be "in the direction".

We changed the sentence according to your advice.

5. In line 364, "cause and result" should be "cause and effect".

We changed the sentence according to your advice.

6. The manuscript occasionally uses "the" instead of "a", or no article. "The" should only be used if it is clear from the context that there is only one of the noun that follows. For example, in line 189, "computations on the networks of N=16 32" should be "computations on networks with N=16 32"

We consulted an English editing service and corrected many expressions accordingly. We attach the certification at the end of this reply.

No data are provided; this is in line with the traditions of reporting the results of simulation-based studies.

Providing the edge lists of the 24 networks whose steady state response is shown in Figure 3 a and b would be an appropriate and informative example of data sharing.

We published codes used for sampling and analysis at Zenodo as DOI: 10.5281/zenodo.3716026. All the data used in the study are also made available there. We added a "Source codes and data availability" subsection after the method section as follows:

> The source codes and the obtained data sets are available at Zenodo (DOI: 10.5281/zen-

odo.3716026).

We provided structures of all GRNs classified as "toggle switch without a lethal edge" (TSwoLE) in the supplemental material "S1 Figure". We also made structural data of these GRNs available at Zenodo stated above. We added the following paragraph to the "Robustness against mutation" subsection in the "Results" section:

> Among these 17 GRNs, 13 were toggle switches and the rest four were one-way switch. This ratio of the toggle switches is significantly higher than that for all the GRNs in the fittest ensemble. We show the network structures of all 13 such GRNs in S1 Figure. We call them "toggle switches without a lethal edge" (TSwoLE).

CERTIFICATE OF ENGLISH EDITING

This document certifies that the paper listed below has been edited to ensure that the language is clear and free of errors. The edit was performed by professional editors at Editage, a division of Cactus Communications.The intent of the author's message was not altered in any way during the editing process. The quality of the edit has been guaranteed, with the assumption that our suggested changes have been accepted and have not been further altered without the knowledge of our editors.

Title Of The Paper

Emergence of cooperative bistability and robustness in gene regulatory networks

Authors

S. Nagata and M. Kikuchi

Job Code

MACI_5

Signature

Vikas Narang,
Chief Operating Officer,
Editage

Date of Issue
March 25, 2020

Editage, a brand of Cactus Communications, offers professional English language editing and publication support services to authors engaged in over 500 areas of research. Through its community of experienced editors, which includes doctors, engineers, published scientists, and researchers with peer review experience, Editage has successfully helped authors get published in internationally reputed journals. Authors who work with Editage are guaranteed excellent language quality and timely delivery.

INFORMATION SECURITY MANAGEMENT SYSTEM DNV·GL ISO/IEC 27001

CACTUS.

Contact Editage

Worldwide	Japan	Korea	China	Brazil	Taiwan
request@editage.com	submissions@editage.com	submit-korea@editage.com	fabiao@editage.cn	contato@editage.com	submitjobs@editage.com
+1 877-334-8243	+81 03-6868-3348	1544-9241	400-005-6055	0800-892-20-97	02 2657 0306
www.editage.com	www.editage.jp	www.editage.co.kr	www.editage.cn	www.editage.com.br	www.editage.com.tw
